# Development, implementation, and evaluation of a registry for the documentation and analysis of assisted suicide in Germany (RegAS) – study protocol

**DOI:** 10.1186/s12910-026-01591-4

**Published:** 2026-09-08

**Authors:** Kerstin Kremeike, Farina Hodiamont, Christina Koenes, Katharina König, Ute Lewitzka, Maria Meinhardt, Jonas Wifek, Irene Schmidtmann, Veronika Dunkl, Alina Kasdorf, Raymond Voltz, Martin Neukirchen, Jacqueline Schwartz, Marc Stefaniak, Barbara Schneider, Reinhard Lindner, Georg Fiedler, Claudia Bausewein, the RegAS Study Group

**Affiliations:** 1 Department of Palliative Medicine, LMU University Hospital, LMU Medizin, Ludwig-Maximilians-Universität München, Marchioninistraße 15, 81377 Munich, Germany; 2 Werner-Felber-Institute for Suicide Prevention and Interdisciplinary Research in Health Care, Dresden, Germany; 3 Department of Psychiatry, Psychosomatic Medicine and Psychotherapy, Goethe-University of Frankfurt, Frankfurt, Germany; 4 Department of Psychiatry and Psychotherapy, Faculty of Medicine and University Hospital Carl Gustav Carus, TUD Dresden University of Technology, Dresden, Germany; 5 Institute of Medical Biostatistics, Epidemiology and Informatics (IMBEI), Johannes Gutenberg University Mainz, Mainz, Germany; 6 Department of Palliative Medicine, University Hospital Cologne, University of Cologne, Cologne, Germany; 7 Interdisciplinary Centre of Palliative Care, Medical Faculty, University Hospital Düsseldorf, Heinrich Heine University Düsseldorf, Düsseldorf, Germany; 8 Department of Anesthesiology, Medical Faculty, University Hospital Düsseldorf, Heinrich Heine University Düsseldorf, Düsseldorf, Germany; 9 National Suicide Prevention Programme (NaSPro), Kassel, Germany; 10 LVR Clinic Cologne Merheim, Cologne, Germany; 11 Department of Social Work, University of Kassel, Kassel, Germany; 12 German Academy for Suicide Prevention (Deutsche Akademie Für Suizidprävention, DASP), Kassel, Germany

**Keywords:** Assisted suicide, Suicide prevention, Registry, Medical ethics, Mixed-methods study, Implementation research, Germany, (health) care research, Medical aid in dying

## Abstract

**Background:**

In 2020, the German Federal Constitutional Court declared the criminal prohibition of assisted suicide as unconstitutional, thereby abolishing previous restrictions. Since then, requests and cases have increased steadily. To ensure transparency and traceability of national assisted suicide practices, systematic and reliable data are required. However, no comprehensive registry currently exists to document and analyze assisted suicide cases in Germany, resulting in a significant data gap for epidemiology, health reporting, and healthcare development. The development, implementation, and evaluation of such a registry is essential to better understand current practices, identify challenges, and improve prevention and support strategies.

**Methods:**

Iterative, mixed-methods, design-based implementation study with five work packages: 1. conceptual, ethical, and legal development of registry content through a scoping review and a group Delphi process; 2. establishment of the legal, ethical, and technical framework of the registry using an iterative, design-based approach; 3. evaluating and refining the registry through a formative mixed-methods design combining quantitative user surveys and web analytics with qualitative think-aloud and retrospective interviews; 4. exploring barriers to the adoption, continuation, and use of the registry through quantitative surveys and in-depth qualitative interviews with potential users; 5. strategic development, dissemination, and integration of the registry within existing healthcare and suicide prevention structures. The study involves expert consultations and stakeholder engagement and is conducted by a multidisciplinary consortium of palliative care and suicide prevention experts in Germany.

**Discussion:**

The establishment of a permanent registry for assisted suicide could substantially improve transparency and empirical knowledge in a highly sensitive and ethically complex field. Systematic documentation may help identify needs, vulnerabilities, and structural challenges faced by individuals considering assisted suicide. In the long term, the findings derived from the registry are expected to contribute to the development of targeted support services in Germany. The study results may also provide valuable insights for other countries facing comparable ethical, legal, and regulatory challenges.

**Trial registration:**

Registered on 25 October 2025 in the BQS (Institute for Quality and Patient Safety) registry, No. 2020467 and on 29 April 2026 in the German Clinical Trials Register (DRKS), No. DRKS00040174.

**Supplementary Information:**

The online version contains supplementary material available at https://doi.org/10.1186/s12910-026-01591-4.

## Background

In 2020, the German Federal Constitutional Court declared Sect. 217 of the Criminal Code, which had prohibited commercial assisted suicide, to be unconstitutional [1, 2]. The Court emphasized that the right to a self-determined death includes the freedom to seek and accept assistance from third parties, regardless of a person’s life situation and not only to people with life-limiting illness [1]. Since then, a steady increase in assisted suicide cases has been reported in Germany [3, 4].

Overall, suicide rates in Germany have declined markedly since the 1980 s but have risen again since 2019, reaching 10,304 deaths in 2023 and increasing further to 10,372 in 2024 [5]. However, official mortality statistics do not distinguish between assisted and non-assisted suicides, limiting reliable monitoring. Available data from individual sources point to a dynamic development: the largest organization in Germany providing assisted suicide reported 898 assisted deaths in 2025 compared to 623 in 2024, with a continuous rise since 2021 [4]. A study on population-based data from the German federal state of Bavaria also indicate a substantial increase in cases since 2020 [3]. Reported data also highlight significant limitations of the current national data infrastructure, as assisted suicide is neither systematically recorded nor separately coded.

In this context, registries on assisted dying are increasingly recognized as important instruments for transparency, accountability, and ethical oversight, as well as for epidemiological research, health reporting, and healthcare system development [6–8]. In countries where assisted dying is legal, structured reporting systems with mandatory notification and independent review enable systematic monitoring of practice. Comparable approaches exist in other jurisdictions, including Austria (www.ascirs.at) and parts of the United States [9–11], where reporting systems provide detailed insights into the circumstances and developments of assisted suicide. In Germany, however, no dedicated registry currently exists, and comprehensive data on affected individuals and the relevant contextual factors are entirely lacking. Given the substantial clinical, ethical, and health care implications of the Federal Constitutional Court’s ruling on assisted suicide, this absence of systematic evidence is striking.

Existing evidence further suggests that assisted suicide differs from conventional suicide regarding sociodemographic and clinical characteristics. For example, the study from Bavaria suggests that individuals who died by assisted suicide were more likely to be older, female, highly educated, socioeconomically advantaged, and to have psychiatric and neurological disorders, particularly depression, neurodegenerative diseases and dementia [12]. Gender differences are well documented in suicide research, with higher non-assisted suicide rates among men and higher rates of suicide attempts among women [13, 14]. In contrast, available data from countries with assisted suicide suggests a more balanced gender distribution [11, 15]. For conventional suicide, a range of additional risk factors has been identified, including gender identity and sexual orientation, with elevated risks among LGBTQI + populations. However, comparable evidence for assisted suicide is largely lacking, highlighting an important gap in knowledge and the need to identify relevant risk groups [16]. Overall, no reliable national data on assisted suicide cases is currently available. Nevertheless, some right-to-die organizations provide descriptive case statistics that, although not independently verifiable, may offer preliminary insights into the motives and circumstances associated with assisted suicide [4].

Against this background, the present study aims to develop, implement, and evaluate a permanent registry for assisted suicide in Germany. Such a registry may enable systematic data collection, improve the empirical basis for ethical and policy debates, and support the development of targeted services and support strategies by identifying relevant risk groups, needs, and patterns over time.

## Methods/design

**Fig. 1:**
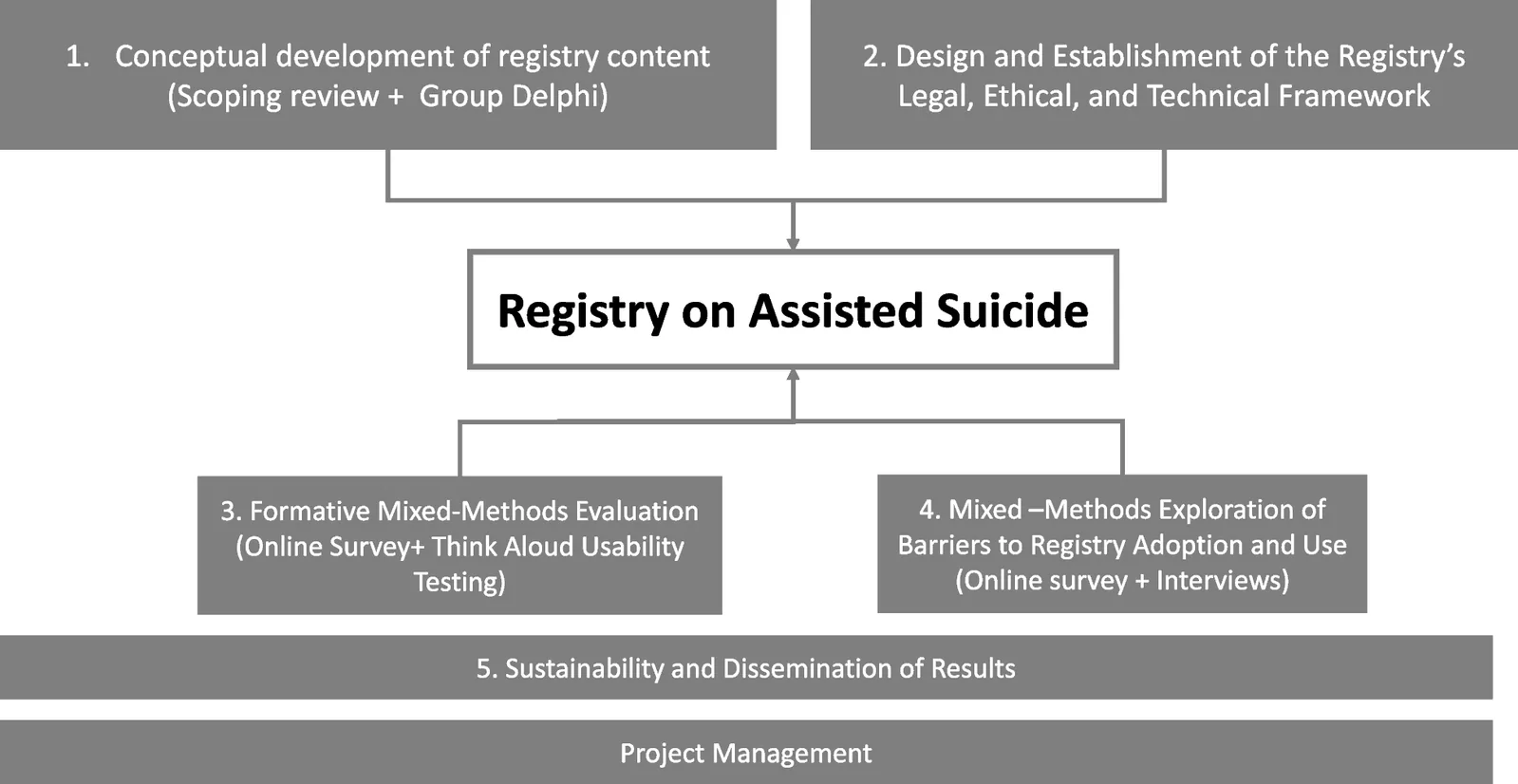
Overview of the study work packages

This study is designed as a 30-month iterative, mixed-methods, design-based implementation project integrating 1. conceptual development, 2. the establishment of an ethical, legal, and technical framework, 3. formative evaluation, 4. exploration of barriers to registry adoption as well as 5. strategic dissemination and integration activities to support the sustainable development and implementation of a national registry for assisted suicide in Germany (see Fig. 1).

### Objectives

The primary objective of this study is to develop, implement, and evaluate a national registry for assisted suicide in Germany.

Secondary objectives are:

1. to establish a consensus-based dataset for the systematic documentation of assisted suicide;
2. to develop a GDPR-compliant ethical, legal, and technical registry infrastructure;
3. to evaluate usability, feasibility, and acceptability of the registry;
4. to identify barriers to registry adoption and sustained use;
5. to support long-term implementation and integration into national suicide prevention and health monitoring structures.

### Outcomes

The primary outcomes of the study are:

- successful development and implementation of a functional registry platform;
- usability and feasibility of registry use.

Secondary outcomes include:

- stakeholder acceptance,
- completeness and quality of collected data,
- barriers to registry adoption,
- usage patterns,
- and sustainability indicators for long-term implementation.

The study is conducted in cooperation with leading institutions in palliative care (e.g. the German Association for Palliative Medicine, a scientific, multi-professional association with over 6,800 members) and in suicide prevention (e.g. the National Suicide Prevention Programme (NaSPro), a nationwide expert network dedicated to suicide, suicidality, and suicide prevention; and the German Society for Suicide Prevention (DGS), the national professional society for suicidology and suicide prevention in Germany, representing and connecting individuals and institutions active in research, teaching, and practice). Other collaborators include the Austrian Palliative Society (OPG; sponsor of the ASCIRS database), and the German Society for Humane Dying (DGHS) as representative of German euthanasia organizations.

The project team brings together interdisciplinary expertise in palliative care and suicidology. It includes leading experts with extensive research experience, as well as a strong track record in developing and maintaining suicide-related registries. Members of the project team have contributed to national clinical guidelines on end-of-life care [17] and dealing with desire to die [18], and have conducted research on assisted suicide requests and related attitudes among healthcare professionals [19–23]. In addition, the team has experience in the development and evaluation of digital platforms [24] and in methodological aspects of registry research [25], including the assessment of data completeness.

Building on this interdisciplinary expertise, the study is structured into five work packages that guide the development, implementation, and evaluation of the registry.

Figure 1 illustrates the overall structure of the project and its interconnected work packages. Table 1 provides an overview of the main project phases and activities across the 30-month study period.

**Table 1:** Overview of project phases and main activities

**Project phase**	**Main activities**
Conceptual development and consensus process (Q1–Q3)	Scoping review and Delphi process for registry development
Technical development and formative evaluation (Q2–Q7)	Development, pilot testing, usability evaluation, and optimization of the registry
Implementation, dissemination, and sustainability (Q6–Q10)	Exploration of barriers to registry use, dissemination activities, and sustainability planning

### Conceptual development of registry content

To establish the basis for the content design of the assisted suicide registry, a scoping review will be conducted to identify, evaluate, and define potential categories and variables. These will then be consented and finalized in a group Delphi process.

#### Scoping review

International databases (MEDLINE, Embase, PsycInfo) will be searched using a predefined strategy combining terms such as assisted suicide, assisted dying, medical aid in dying, registry, database, report, and documentation. The search will be limited to publications from the last ten years (2015–2025) to capture current documentation practices, registries, and databases. In addition, country-specific websites and reports on assisted suicide documentation will be reviewed, including sources from Germany, Austria [26], Switzerland [27], Belgium [28], the Netherlands [29], Spain [30], Canada [31], Oregon (USA) [11], Columbia [32], Australia [33, 34], and New Zealand [35].

From eligible publications and reports, data will be extracted on (1) characteristics of persons who made use of assisted suicide (e.g. age, gender, underlying conditions, reasons for unbearable suffering such as uncontrolled pain, loss of autonomy, functional decline, psychological distress, or loss of dignity), (2) circumstances of assisted suicide (e.g. location, substances used, mode of administration, measures to ensure fulfillment of legal regulations), and (3) characteristics of those providing assistance (e.g. professional background and qualifications). Extracted variables will be organized in a structured matrix. The scoping review will be conducted in accordance with the PRISMA Extension for Scoping Reviews (PRISMA-ScR) [36]. Based on the findings, a preliminary set of candidate variables for inclusion in the national assisted suicide registry will be developed, covering relevant sociodemographic and contextual factors, including gender identity beyond binary categories. This set will be then refined through the subsequent Delphi process.

#### Consensus process using the group Delphi method

Consensus on registry variables will be established using a modified Delphi approach comprising an initial online survey and a subsequent two-day in-person Delphi workshop. The online survey will assess agreement on the proposed variables in a broader panel of experts and identify those items for which consensus has already been achieved. Variables meeting the predefined consensus criteria will be excluded from workshop discussion, enabling a more focused and efficient deliberation of unresolved items while preserving broad expert input.

The group Delphi workshop will involve three to four groups of four to five participants each (*n* = 16–20) [37]. Participants will be purposively selected according to predefined criteria, including professional background, affiliation with relevant organizations, experience with assisted suicide, and involvement as affected relatives, to ensure relevance and applicability. The workshop will be moderated by an independent professional facilitator. Through structured group discussion and voting, individual contributions will be systematically documented, transparently synthesized, and integrated into a final consensus-based variable set for the registry. This approach addresses a known limitation of the traditional Delphi method, in which experts may be reluctant to revise their views based solely on aggregated feedback. The Delphi process will follow the Conducting and Reporting of Delphi Studies (CREDES) recommendations [38].

#### Design and establishment of the registry’s legal, ethical, and technical framework

The registry’s legal, ethical, and technical framework defines the procedures for data collection, processing, and analysis, ensuring data quality, privacy protection, and compliance with regulatory requirements. Therefore, a comprehensive data protection and processing framework will be developed in accordance with the General Data Protection Regulation (GDPR), which constitutes the primary legal basis for this project. This framework defines the required data elements, data minimization strategies, governance structures, cooperation agreements, risk assessments, and organizational and administrative responsibilities. It will also specify the technical infrastructure and procedures for secure data handling and quality assurance.

#### Data collection and technical infrastructure

Data collection is conducted through a secure, web-based questionnaire. Data may be entered into the registry by individuals who have directly participated in or witnessed an assisted suicide (e.g., physicians, family members, representatives of assisted dying organizations), as well as by individuals who considered assisted suicide but discontinued the process. All data is fully anonymized upon entry, ensuring that no individual can be re-identified. Thus, no IP addresses are stored or recorded as direct identifiers of the persons entering the data. Participants are not required to provide personal information. Data are stored on encrypted servers in Germany, with access restricted to authorized project team members. Data is processed exclusively for research purposes. Anonymized data may only be shared with third parties for scientific research, and only after formal review and approval by the project’s steering committee. Such sharing is limited to qualified research institutions and individual researchers and is subject to strict data use agreements.

The content of the registry developed in the first work package captures key information on demographics, motivations, procedural aspects and contextual factors. Once collected, data undergoes a structured processing pipeline. First, data cleaning is performed to identify and resolve inconsistencies, missing values and outliers. This includes automated checks for logical coherence (e.g. age vs. age-related disease) and manual review (e.g. to delete personal information given in a text field).

#### Pilot testing, validation and iterative refinement

Following the development of the technical infrastructure, the registry will be piloted with a group of potential users (e.g. physicians and representatives of assisted dying organizations). During this phase, real cases (where ethically permissible) and theoretical case vignettes will be documented to assess feasibility, usability, and data quality. This approach allows early identification of technical and procedural challenges and supports optimization prior to full implementation.

Based on the findings from the pilot phase and validation procedures, iterative technical and content-related refinements of the registry will be implemented. This includes adjustments to the questionnaire design, data entry interface, and underlying data processing framework to improve usability, data quality, and completeness.

#### Data processing

The cleaned and validated dataset is then used for statistical analysis. Descriptive statistics, trend analyses and subgroup comparisons are conducted to explore patterns in motivations, demographics and procedural aspects. Multivariate models are applied to identify associations between variables with the overarching aim of identifying trends, regional differences and risk profiles among individuals considering or undergoing assisted suicide, ultimately contributing to the development of a comprehensive national registry on assisted suicides and evidence-based support strategies for vulnerable groups.

Data validity and completeness will be assessed through comparison with external data sources, such as death certificates, and by applying capture–recapture methods to estimate potential underreporting.

### Formative mixed-methods evaluation of the registry

To assess usability, acceptability, and feasibility, a formative mixed-methods evaluation will be conducted. Combining quantitative and qualitative approaches enables the capture of both user satisfaction and in-depth user experiences and is well established in the evaluation of digital health platforms [39].

#### Quantitative component of the evaluation

An online survey will be integrated into the registry platform. After completing registration, users will be invited via a pop-up window to participate in a short online survey in which they can voluntarily and anonymously evaluate their satisfaction with, and perceptions of the user-friendliness, the perceived clarity, and efficiency of data entry. Data security and sustainability aspects are considered just as much as the actual motivation for using the register. The target sample size is approximately 50 to 100 respondents. In addition, an integrated analysis tool will be used to collect aggregated web statistics (e.g. time required) to evaluate effectiveness and usage patterns.

#### Qualitative component of the evaluation

A sample of five to ten users will participate in structured usability testing sessions conducted via video conferencing. Participants will use the “think-aloud” method while using the platform, verbalizing their thoughts, expectations, and difficulties in real time. The sessions will be audio-recorded and transcribed verbatim. These brief semi-structured interviews will explore participants ‘overall impressions and perceived barriers.

All data will be pseudonymized prior to analysis.

### Mixed-methods exploration of barriers to registry adoption and use

To assess barriers to use and reasons for discontinuation or non-use of the register, quantitative and qualitative findings will be integrated using mixed-methods triangulation, allowing a comprehensive understanding [40–42]. This approach aims to identify key barriers, cross-cutting patterns, and opportunities to modify both, the communication strategy and the register itself, thereby improving the completeness and quality of the collected data.

#### Quantitative survey

An online survey will be developed to capture barriers and reasons for discontinuing or avoiding register use. The survey is exploratory in nature and comprises five closed questions with 5-point Likert-scale response options and two open-ended questions. Items address different dimensions (e.g., ethical, legal) and subjectively perceived barriers.

Participation is anonymous, respondents may optionally provide sociodemographic information. The survey is accessible via a pop-up link within the register and permanently via the register’s website. A sample of approximately 20–30 surveys is anticipated. In line with methodological references, small sample sizes are considered sufficient to capture the majority of usability- and acceptance-related issues within clearly defined user groups [43, 44].

Closed-ended items will be analyzed descriptively. Open-ended responses will be contextualized using qualitative content analysis [45].

#### Qualitative interviews

Semi-structured interviews will be conducted to enable in-depth exploration. Participants include healthcare professionals and other providers or supporters of assisted suicide who do not use the register, have discontinued an entry, or wish to express reservations regarding register use. Semi-structured interviews are particularly suitable for exploring sensitive topics and subjective decision-making processes [46].

Interviews will be conducted by telephone or online. Recruitment will take place via the register’s website as well as through relevant assisted-suicide organizations and professional networks. An interview guide comprising four thematic sections (experiences and reception of assisted suicide, reception of a register, barriers and reservations) will be developed by the study team, informed by expert input and piloted in mock interviews.

Interviews will be audio-recorded, transcribed verbatim with complete anonymization of identifying data, and analyzed using reflexive thematic analysis as described by Braun and Clarke [47]. A sample of 10–20 interviews is planned, with recruitment continuing until sufficient thematic depth relevant to the aims of the work package is achieved.

### Sustainability and dissemination of results

The National German Suicide Prevention Strategy underscores the importance of monitoring suicide, including assisted suicide, to identify at-risk groups and guide the development of targeted support services. The development, implementation, and evaluation of a registry for documenting assisted suicides may constitute an important contribution to these monitoring activities. Relevant professional associations support the establishment of such a national registry. To ensure sustainability beyond the funding period, the registry is intended to be hosted by the Werner Felber Institute, a research institution specializing in suicidology with experience in managing sensitive health-related databases and with the infrastructure required for secure data storage and compliance with applicable data protection standards.

The project will be communicated to both professional and public audiences, building on the established dissemination structures of the NaSPro. Planned activities include the development of informational materials and a dedicated website, consultations with organisations involved in assisted suicide to optimise outreach, targeted communication through professional medical societies, and publications in general medical journals to ensure broad awareness among healthcare professionals.

### Project management

The project lead will provide overall coordination of the consortium and support the project partners in the implementation of their respective work packages. Monthly video conferences involving all partners will be held throughout the project period, complemented by additional exchanges between individual work packages or smaller groups as needed.

A key responsibility of project management will be to ensure the timely achievement of work package objectives as well as to facilitate effective and mutually beneficial exchange across work packages. In addition, the project management team will coordinate the advisory board and convene regular meetings.

### Advisory board

To ensure relevance, transparency, and stakeholder-informed decision-making, an advisory board will be established in line with recommendations for stakeholder involvement in health and implementation research [48, 49]. The board will provide ongoing guidance throughout the project and will include representatives with experience in the Austrian reporting and learning system for assisted suicide practice (ASCIRS), representatives of assisted suicide organizations including the DGHS, as well as experts in palliative care, suicide prevention, medical ethics, and registry research from German and Austria. Media representatives and individuals advocating access to assisted suicide will also be included to reflect a broad range of perspectives.

The composition of the advisory board will be determined by the project team at the outset of the project. The board will meet virtually two to three times over the course of the project to review progress and discuss key milestones, including completion of the registry’s conceptual and technical development, evaluation of initial findings, and potential refinements to the registry.

### Reporting

The goal is to establish a high-quality and permanently maintained national database for assisted suicides in Germany. The Werner Felber Institute will ensure the long-term usability of the registry data and issue annual reports. These reports may inform the development of the recommended support services. The first registry report, analysing assisted suicides, will be presented and discussed at an (online) event during the project period, and all reports will be made available via the registry’s online platform. In the medium term, a linkage with the German Federal Health Monitoring system will also be explored. As a registry is likely to be established as part of the regulatory process concerning assisted suicide, sustainable funding may become available. In the long term, the annual reports of the registry could be integrated into the federal health reporting of the Robert Koch Institute (RKI) to support public health monitoring. During the project period, contact with the RKI will therefore be established.

## Discussion

This study protocol describes the development, implementation, and evaluation of a national registry for assisted suicide in Germany, addressing a highly sensitive and ethically complex field. In the absence of comprehensive nationwide data, the registry is expected to provide a systematic and transparent empirical basis to better understand current practices, inform ethical and policy debates, support suicide prevention efforts, and generate data for epidemiological research, health reporting, and healthcare services research.

Building on international experience, registries on assisted dying enable systematic monitoring, regulatory oversight, and transparent analysis of trends [50]. Evidence from countries with established reporting systems suggests that standardised documentation enhances transparency and supports consistent practice. By adapting these approaches to the German context and integrating expert and stakeholder perspectives, this study aims to develop a context-sensitive and sustainable registry model.

A key strength of this study lies in its iterative, mixed-methods, design-based implementation approach, which integrates conceptual development, stakeholder engagement, and real-world testing, consistent with current guidance on complex interventions and implementation science [51, 52]. The combination of a scoping review and a group Delphi process ensures that the registry content is grounded in both empirical evidence and expert consensus. The involvement of a multidisciplinary project team further enhances the relevance and applicability of the registry across clinical, ethical, and public health contexts. In addition, pilot testing and formative evaluation enable continuous refinement of the registry and support the development of a user-oriented and methodologically robust system.

Several limitations should be considered. As participation in the registry will be initially voluntary, underreporting is possible, and the data may be subject to selection bias. Certain groups or settings may be over- or underrepresented, potentially limiting the generalisability of findings. Individuals and organisations with positive or negative attitudes towards assisted suicide may differ systematically in their willingness to participate in the registry. Furthermore, the reliance on self-reported and third-party data may affect data completeness and accuracy. Although strategies such as data validation and comparison with external sources are planned, a tension remains between ensuring strict data protection and enabling comprehensive data validation [53–55].

The registry is situated within a complex ethical landscape. Assisted suicide raises fundamental questions regarding autonomy, vulnerability, and the role of healthcare systems [7]. Key ethical challenges include protecting sensitive data, preventing misuse or misinterpretation of findings, and avoiding unintended normalisation of assisted suicide practices [56]. These challenges are addressed through a GDPR-compliant data protection framework, robust governance structures, and careful consideration of data interpretation and dissemination. The registry is designed as a descriptive and analytical tool rather than a normative instrument. It is not intended to promote or facilitate assisted suicide, but rather to provide transparent empirical data to support ethical reflection, suicide prevention, public health monitoring, and evidence-informed policy discussions.

The findings of this study are expected to be relevant for policy, clinical practice, and future research. By providing systematic data on assisted suicide practices, the registry may support evidence-informed policy development and ongoing ethical and legislative debates. In clinical practice, identifying patterns, needs, and risk groups may inform targeted support and prevention strategies. For research, the registry provides a foundation for analyzing trends and contextual factors and may serve as a model for other countries facing similar challenges.

## Supplementary Information

Supplementary Material 1.

## Data Availability

No datasets were generated or analysed during the current study.

## Abbreviations

ASCIRS: Austrian Suicide and Assisted Suicide Reporting and Information Registry System
BMG: German Federal Ministry of Health (Bundesministerium für Gesundheit)
CREDES: Conducting and Reporting of Delphi Studies
DASP: German Academy for Suicide Prevention (Deutsche Akademie für Suizidprävention)
DGHS: German Society for Humane Dying (Deutsche Gesellschaft für Humanes Sterben)
DGP: German Association for Palliative Medicine
DGS: German Society for Suicide Prevention
DRKS: German Clinical Trials Register
GDPR: General Data Protection Regulation
IMBEI: Institute of Medical Biostatistics, Epidemiology and Informatics
LMU: Ludwig Maximilian University
NaSPro: National Suicide Prevention Programme
OPG: Austrian Palliative Society
PRISMA-ScR: Preferred Reporting Items for Systematic Reviews and Meta-Analyses Extension for Scoping Reviews
RKI: Robert Koch Institute
RECORD: REporting of studies Conducted using Observational Routinely-collected health Data
RegAS: Registry for the documentation and analysis of assisted suicide in Germany
STROBE: Strengthening the Reporting of Observational Studies in Epidemiology
