# Supplementary material for: Development, implementation, and evaluation of a registry for the documentation and analysis of assisted suicide in Germany (RegAS) – study protocol
Source: BMC Med Ethics. 2026 Sep 8;27:190. doi: 10.1186/s12910-026-01591-4 (PMC13551708; doi:10.1186/s12910-026-01591-4)
Supplement: Supplementary file 1 [file 12910_2026_1591_MOESM1_ESM.docx]

# SPIRIT 2013 Checklist – RegAS Study Protocol

Development, implementation, and evaluation of a registry for the documentation and analysis of assisted suicide in Germany (RegAS) – study protocol

| Section/item | Item No. | Description | Addressed in protocol |
| --- | --- | --- | --- |
| Title | 1 | Descriptive title identifying the study design, population, interventions, and trial acronym | Title page |
| Trial registration | 2a | Trial identifier and registry name | Abstract – Trial registration |
| Trial registration | 2b | WHO Trial Registration Data Set | DRKS registration available |
| Protocol version | 3 | Date and version identifier | Title page |
| Funding | 4 | Sources and types of financial support | Funding section |
| Roles and responsibilities | 5a | Names, affiliations, and roles of protocol contributors | Title page / Authors’ contributions |
| Roles and responsibilities | 5b | Sponsor contact information | Funding section |
| Roles and responsibilities | 5c | Role of sponsor/funder | Funding section |
| Roles and responsibilities | 5d | Committees/groups overseeing study conduct | Advisory Board / Project Management |
| Background and rationale | 6a | Research question and justification | Background |
| Background and rationale | 6b | Explanation for choice of comparators | Not applicable |
| Objectives | 7 | Specific objectives and hypotheses | Methods/Design – Objectives |
| Trial design | 8 | Description of study design | Methods/Design |
| Study setting | 9 | Description of study settings | Methods/Design |
| Eligibility criteria | 10 | Inclusion and exclusion criteria | Work packages 1–4 |
| Interventions | 11a | Description of interventions/processes | Registry development and evaluation procedures |
| Interventions | 11b | Criteria for discontinuing interventions | Voluntary participation |
| Interventions | 11c | Strategies to improve adherence | Stakeholder involvement and usability optimization |
| Interventions | 11d | Relevant concomitant care/interventions | Not applicable |
| Outcomes | 12 | Primary and secondary outcomes | Methods/Design – Outcomes |
| Participant timeline | 13 | Schedule of enrolment, interventions, assessments | Table 1 |
| Sample size | 14 | Estimated sample sizes and rationale | Work packages 2–4 |
| Recruitment | 15 | Recruitment strategies | Methods sections for work packages |
| Data collection methods | 18a | Plans for data collection and assessment | Data collection and evaluation sections |
| Data collection methods | 18b | Retention and follow-up procedures | Registry continuation procedures |
| Data management | 19 | Data entry, coding, security, storage | Legal, ethical and technical framework |
| Statistical methods | 20a | Statistical analysis methods | Data Processing section |
| Statistical methods | 20b | Additional analyses | Trend analyses and subgroup analyses |
| Statistical methods | 20c | Handling of missing data | Data cleaning procedures |
| Data monitoring | 21a | Data monitoring committee | Steering committee / advisory structures |
| Data monitoring | 21b | Interim analyses and stopping guidelines | Not applicable |
| Harms | 22 | Collection and management of adverse events | Ethical safeguards and support procedures |
| Auditing | 23 | Auditing procedures | Governance framework |
| Research ethics approval | 24 | REC/IRB approval | Ethics approval section |
| Protocol amendments | 25 | Communication of modifications | Governance procedures |
| Consent or assent | 26a | Informed consent procedures | Consent for publication / methods |
| Consent or assent | 26b | Additional consent provisions | Not applicable |
| Confidentiality | 27 | Protection of personal data | GDPR framework section |
| Declaration of interests | 28 | Financial and other competing interests | Competing interests |
| Access to data | 29 | Who will have access to data | Data sharing procedures |
| Ancillary and post-trial care | 30 | Ancillary care arrangements | Not applicable |
| Dissemination policy | 31a | Plans for communication of results | Sustainability and dissemination |
| Dissemination policy | 31b | Authorship eligibility | Authors’ contributions |
| Dissemination policy | 31c | Public access to protocol/data | Availability of data and materials |
| Informed consent materials | 32 | Model consent forms | To be added as supplementary material if required |
| Biological specimens | 33 | Collection/storage of biological specimens | Not applicable |

## Notes on SPIRIT applicability

This protocol describes a complex mixed-methods implementation and registry development study rather than a randomized controlled trial. Therefore, several SPIRIT items related to allocation, blinding, and intervention assignment are not applicable.

The checklist has therefore been adapted to reflect the methodological characteristics of the RegAS project, including registry development, implementation research, usability evaluation, qualitative and quantitative mixed-methods approaches, stakeholder involvement, and governance and ethical oversight procedures.
